# microRNA dependent and independent deregulation of long non-coding RNAs by an oncogenic herpesvirus

**DOI:** 10.1371/journal.ppat.1006508

**Published:** 2017-07-17

**Authors:** Sunantha Sethuraman, Lauren Appleby Gay, Vaibhav Jain, Irina Haecker, Rolf Renne

**Affiliations:** 1 Department of Molecular Genetics and Microbiology, University of Florida, Gainesville, Florida, United States of America; 2 UF Health Cancer Center, University of Florida, Gainesville, Florida, United States of America; 3 UF Genetics Institute, University of Florida, Gainesville, Florida, United States of America; University of North Carolina at Chapel Hill, UNITED STATES

## Abstract

Kaposi’s sarcoma (KS) is a highly prevalent cancer in AIDS patients, especially in sub-Saharan Africa. Kaposi’s sarcoma-associated herpesvirus (KSHV) is the etiological agent of KS and other cancers like Primary Effusion Lymphoma (PEL). In KS and PEL, all tumors harbor latent KSHV episomes and express latency-associated viral proteins and microRNAs (miRNAs). The exact molecular mechanisms by which latent KSHV drives tumorigenesis are not completely understood. Recent developments have highlighted the importance of aberrant long non-coding RNA (lncRNA) expression in cancer. Deregulation of lncRNAs by miRNAs is a newly described phenomenon. We hypothesized that KSHV-encoded miRNAs deregulate human lncRNAs to drive tumorigenesis. We performed lncRNA expression profiling of endothelial cells infected with wt and miRNA-deleted KSHV and identified 126 lncRNAs as putative viral miRNA targets. Here we show that KSHV deregulates host lncRNAs in both a miRNA-dependent fashion by direct interaction and in a miRNA-independent fashion through latency-associated proteins. Several lncRNAs that were previously implicated in cancer, including MEG3, ANRIL and UCA1, are deregulated by KSHV. Our results also demonstrate that KSHV-mediated UCA1 deregulation contributes to increased proliferation and migration of endothelial cells.

## Author summary

KS is the most prevalent cancer associated with AIDS in sub-Saharan Africa, and is also common in males not affected by AIDS. KSHV manipulates human cells by targeting protein-coding genes and cell signaling. Here we show that KSHV alters the expression of hundreds of human lncRNAs, a broad class of regulatory molecules involved in a variety of cellular pathways including cell cycle and apoptosis. KSHV uses both latency proteins and miRNAs to target lncRNAs. miRNA-mediated targeting of lncRNAs is a novel regulatory mechanism of gene expression. Given that most herpesviruses encode miRNAs, this mechanism might be a common theme during herpesvirus infections. Understanding lncRNA deregulation by KSHV will help decipher the important molecular mechanisms underlying viral pathogenesis and tumorigenesis.

## Introduction

Kaposi’s sarcoma-associated herpesvirus (KSHV) is an opportunistic human oncovirus, which causes Kaposi’s sarcoma (KS), Primary Effusion lymphoma (PEL) and Multicentric Castleman’s disease (MCD) in immunocompromised individuals, primarily AIDS patients and organ-transplant recipients [1]. KSHV uses the lytic mode of replication for spread of infection, and latency for persistence in the host. All tumor cells isolated from KS patients test positive for latent viral episomes [1]. Latent KSHV expresses only 10% of its 140-kb dsDNA genome, encoding primarily four latency proteins (Kaposin, vFLIP, vCyclin and LANA) and 25 mature miRNAs [1]. miRNAs are 21–23 nt long non-coding RNAs that recognize target mRNAs using 7 bp ‘seed sequences’ and silence them (see [2] for review). To identify the means by which KSHV causes tumors, KSHV latency proteins and miRNAs have been studied extensively [1]. Ribonomics approaches to identify targets of KSHV miRNAs have focused exclusively on mRNAs [3, 4].

Recently, lncRNAs have emerged as important regulatory molecules in cancer [5]. LncRNAs play a variety of regulatory roles in both the cytoplasm and nucleus [6, 7]. This group includes all RNA molecules longer than 200 nt with no apparent coding potential, and they have diverse functions ranging from acting as a scaffold, sponge/decoy or guide aiding in cell-signaling [6, 8]. Owing to their diversity, over 95% of the lncRNAs remain uncharacterized. Disease association is a starting point for identifying and characterizing lncRNAs with important regulatory roles. Using this approach with different cancer types, oncogenic lncRNAs such as MALAT-1, ANRIL, UCA1, and tumor suppressor lncRNAs like Gas-5 and MEG3 have been functionally characterized [5]. Another important group of disease-relevant lncRNAs includes those involved in the innate immune response following viral or bacterial infections [9]. A few studies have addressed the roles of host lncRNAs during viral infections, for example HULC (Hepatitis-B) and NRON (HIV) [10]. However, the question of whether viruses manipulate specific host lncRNAs to their advantage remains largely unexplored. Understanding deregulation of specific host lncRNAs, especially cancer-related lncRNAs by persistent oncoviruses, such as the γ-herpesviruses, would shed light on how these viruses drive oncogenesis.

Regulatory cross-talk is known to occur between miRNAs and lncRNAs, at multiple levels. LncRNAs like BIC1 and H19 act as precursors for miRNAs [11, 12] and lncRNAs such as HULC and CDR1-AS act as sponges for miRNAs [13, 14]. Conversely, human miRNA miR-9 represses the expression levels of the lncRNA MALAT1 [15]. Work from the Steitz laboratory demonstrated that the viral lncRNAs HSUR1 and HSUR2, encoded by Herpesvirus Saimiri, act as sponges for cellular miR-16, miR-142-3p and miR-27 and thereby silence some of these miRNAs in T-lymphocytes, suggesting that γ-herpesviruses can utilize virus lncRNAs to target host miRNAs[16]. Conversely, whether herpesvirus miRNAs can target and downregulate host lncRNAs remains an open question.

In this study, we demonstrate that latent KSHV infection of endothelial cells alters the host lncRNA profile. We provide evidence that KSHV deregulates hundreds of host lncRNAs including many cancer-associated lncRNAs such as UCA1, ANRIL and MEG3 in both a miRNA dependent and independent manner. Furthermore, KSHV appears to manipulate the host lncRNAs to favor proliferation and migration of latently infected endothelial cells.

## Results

### KSHV deregulates host lncRNAs

Previously, we identified the mRNA targetome of viral miRNAs in PEL cells by High Throughput Sequencing-Crosslinking Immuno Precipitation (HITS-CLIP) analysis of the Ago protein [3]. The PEL cell lines we studied were BC-3 and BCBL-1, which are KSHV positive B-cell lines. We reanalyzed the HITS-CLIP data for enriched lncRNAs and compared our results with a similar reinvestigation of Ago PAR-CLIP data from lymphoblastoid cell lines infected with Epstein-Barr Virus (EBV) [17], a related γ-herpesvirus that causes cancer. We found that approximately 357 and 750 lncRNAs were a part of the KSHV and EBV miRNA targetome, respectively, and 64 lncRNAs were potentially targeted by miRNAs from both viruses **(S1 Table)**.

**Fig 1:**
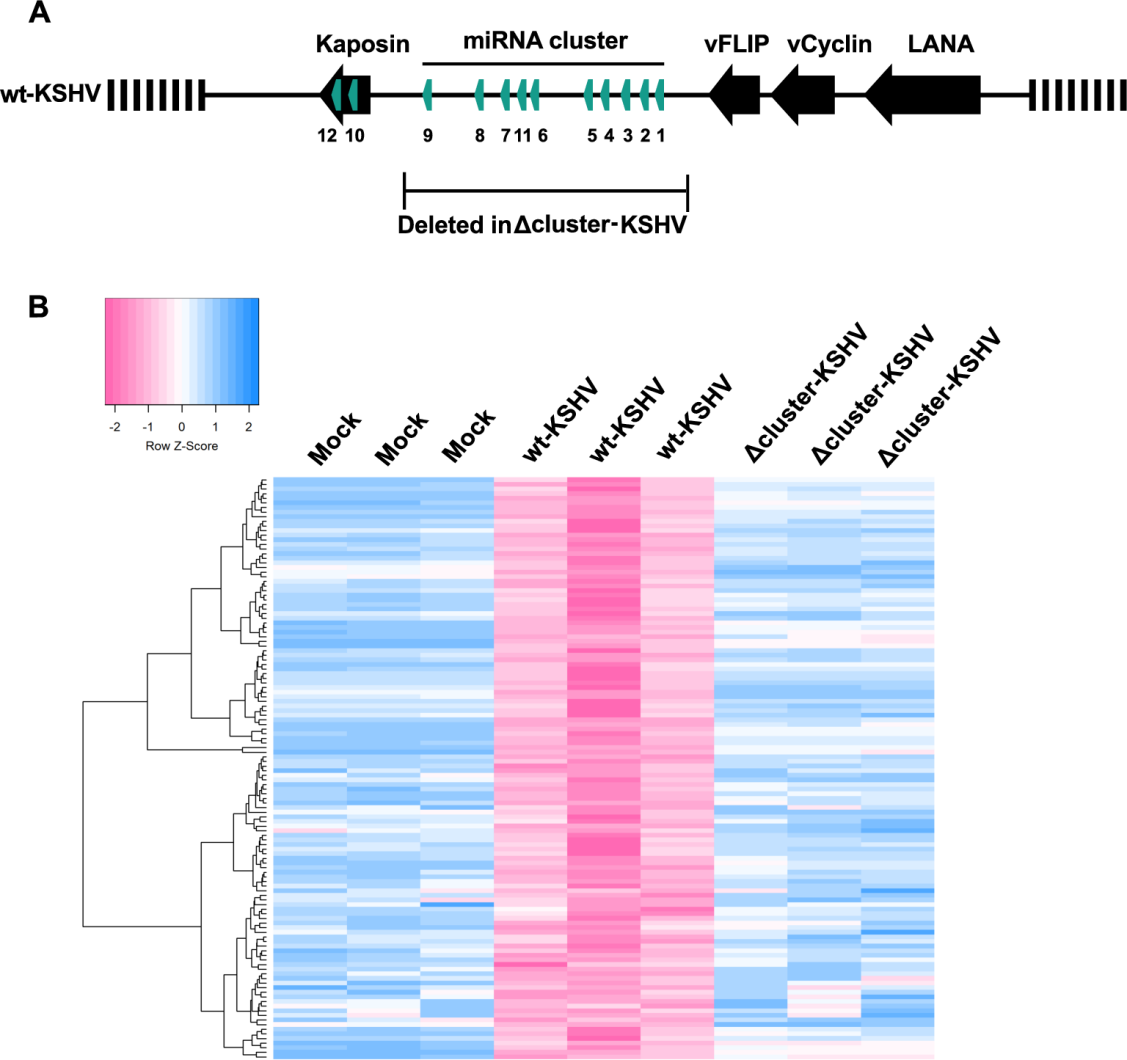
Expression profiling of wt-KSHV and Δcluster-KSHV infected endothelial cells. **(A)** Latency associated region of wt-KSHV in a Bac16 backbone. The region deleted in the Δcluster-KSHV virus is highlighted. **(B)** Heatmap of unsupervised hierarchical clustering of the microarray samples in the ‘rescued’ category of genes (n = 3 technical replicates).

We aimed to determine the effect of latent KSHV infection on the lncRNA expression profile of endothelial cells and specifically question whether KSHV encoded miRNAs targeted endothelial lncRNAs. To address these questions, we used Telomerase Immortalized Vein Endothelial (TIVE) cells, an *in vitro* model system to study KS [18]. We performed lncRNA expression profiling on latently infected TIVE cells harboring either the wt-KSHV or Δcluster-KSHV [19, 20], in which a region containing 10 of the 12 miRNA genes is deleted, and used the lncRNA profile of mock-infected TIVE cells as reference. The KSHV latency-associated region of the wt and mutant bacmid backbones used for this experiment is shown in **Fig 1A**. The profiling analysis revealed that wt-KSHV and Δcluster-KSHV infections deregulate 858 and 2372 host lncRNAs, respectively (**Table 1**), indicating that latent KSHV infection globally affects lncRNA expression. The higher count of deregulated lncRNAs in Δcluster-KSHV infection is likely due to increased spontaneous reactivation rate in the absence of viral miRNAs [20, 21]. The differentially expressed lncRNAs are listed in **S2 Table**. We grouped the deregulated lncRNAs into three categories based on a cut-off of fold change ≥ 2.0: upregulated, downregulated and rescued. We defined rescued genes as those that were downregulated in wt-KSHV-infected cells compared to mock, and were upregulated in Δcluster-KSHV-infected cells compared to wt-infected cells. We validated using qRT-PCR two downregulated lncRNAs, two upregulated lncRNAs, and three rescued lncRNAs that were identified from the microarray analysis (**S1 Fig**). We identified 126 candidates in the rescued category, which are putative direct targets of viral miRNAs (**Fig 1B**).

**Table 1:** Summary of deregulated lncRNAs from the microarray analysis.

	wt-KSHV vs. Mock	Δcluster-KSHV vs. Mock	Common
Upregulated	325	1107	247
Downregulated	533	1265	238

### Mature viral miRNAs and Ago-2 are present in the nuclei of KSHV-infected cells

Based on lncRNA localization data from HUVEC cells [22], at least 9 of the 126 putative lncRNA targets of viral miRNAs we identified are exclusively nuclear localized, and 32 of them are partially nuclear localized. It is important to note that the localization information was available for only 72 out of the 126 rescued lncRNAs. Similarly, the 357 lncRNAs identified from Ago HITS-CLIP of PEL cells include nuclear resident lncRNAs such as ANRIL (CDKN2B-AS1) and MALAT-1. Moreover, several of the uncharacterized candidates of these 357 lncRNAs may be nuclear localized. miRNA-mediated regulation of nuclear localized lncRNAs seemed paradoxical at the outset, as mature miRNAs and RISCs including the Ago family proteins are believed to reside and function in the cytoplasm. Recently, several groups showed that Ago-2 complexes can be present in the nuclei of different cell types [23, 24]. Moreover, studies in Hodgkin’s lymphoma lines identified that several lncRNAs co-isolate with Ago protein [25]. To determine whether KSHV miRNAs could regulate nuclear lncRNAs, we investigated the nuclear/cytoplasmic distribution of viral miRNAs and Ago-2 in PEL cells.

**Fig 2:**
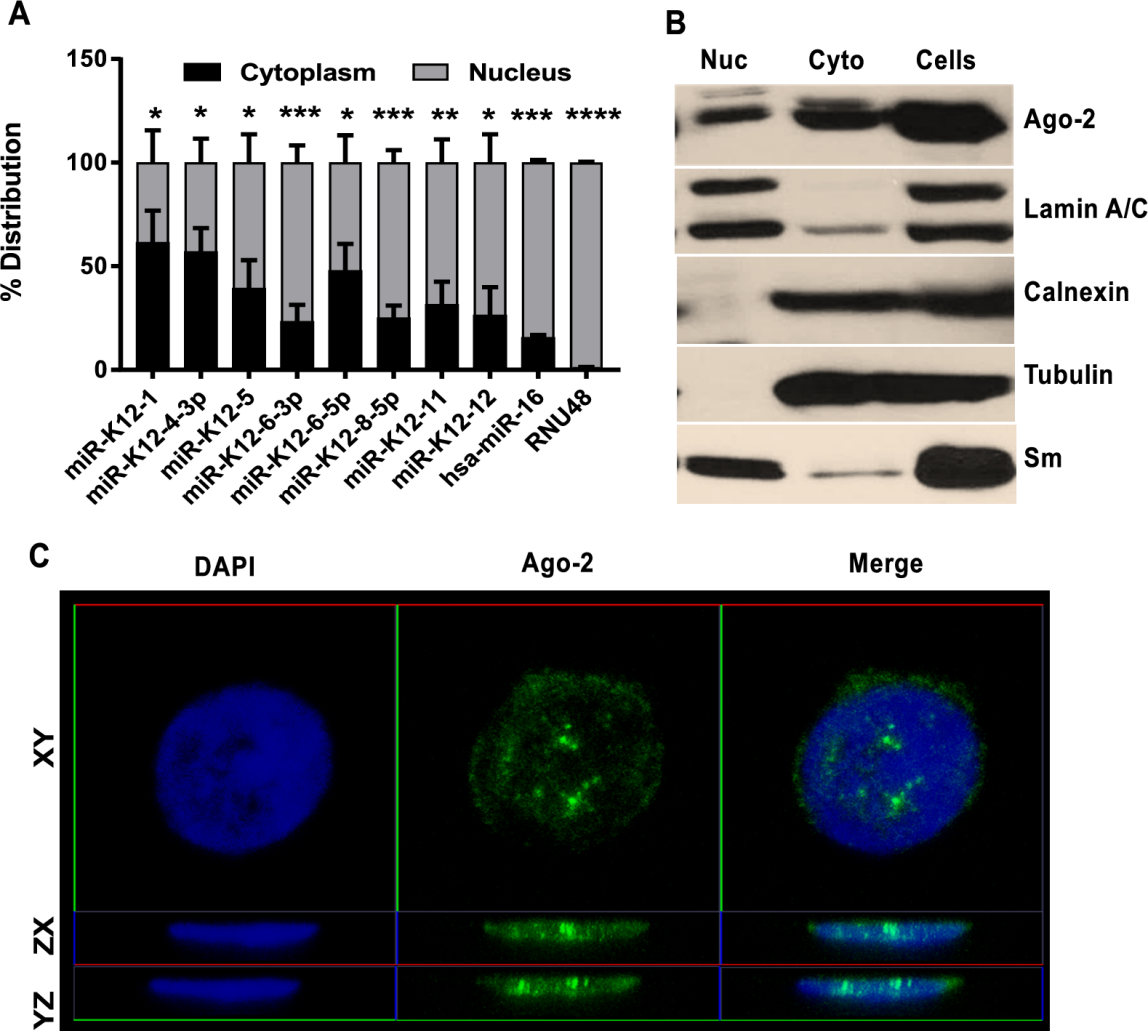
KSHV miRNAs and Ago2 are partially localized in the nuclei of latently infected cells. **(A)** qRT-PCR analysis of mature KSHV miRNA distribution in the cytoplasmic and nuclear fractions of PEL cells. Percentage distribution was calculated by normalizing to expression in whole PEL cells, assuming no loss during fractionation. RNU48 was used as a nuclear control for fractionation. The bar graphs show the mean values (n = 3) ± SEM. p-values: * < 0.05; ** < 0.01; *** < 0.005. **(B)** Subcellular distribution of Ago2 proteins in PEL cells analyzed using Western blotting. Tubulin was probed as positive control for cytoplasm, Sm and Lamin A/C are positive controls for nuclei and Calnexin is the negative control for Endoplasmic Reticulum **(C)** Localization of Ago2 in PEL nuclei analyzed using IFA and confocal microscopy. Ago2 is shown in green and DAPI in blue. DAPI is shown at half the original intensity.

We fractionated BCBL-1 cells into nucleus and cytoplasm and analyzed the distribution of KSHV miRNAs using stem-loop RT-qPCR, which amplifies mature miRNAs but not their precursors. Mature KSHV miRNAs were found in both the cytoplasmic and nuclear fraction (**Fig 2A**). It is important to note that a cellular miRNA hsa-miR-16 is also distributed between the nucleus and the cytoplasm (**Fig 2A**), and such partial nuclear localization of mature miRNAs has been previously reported in other cell lines [26, 27]. We probed the fractions for Ago-2 using western blotting (**Fig 2B**). Calnexin, an ER resident, was used as a control to ensure that the nuclear preparations were free of endoplasmic reticulum. A significant fraction of Ago-2 was localized in the nucleus of BCBL-1 cells. This observation is consistent with a study by Gagnon et al., which reported comparable amounts of Ago2 in the nucleus and cytoplasm of multiple cell lines including HeLa, T47D, A549 and fibroblasts [23]. These results were confirmed using immunofluorescence analysis (IFA) of Ago-2 in isolated BCBL-1 nuclei by confocal microscopy and 3D-reconstruction. The images in **Fig 2C** show Ago-2 in all planes of view (XY, YZ and ZX) with and without DAPI, and it is evident that Ago-2 is present inside the BCBL-1 nuclei. We observed similar results with IFA performed on KSHV-infected TIVE cells (**Movie S1**). Thus, we concluded that Ago2 and viral miRNAs are present in the nuclei of infected cells, and miRNAs could potentially interact via Ago2 with nuclear lncRNAs.

### KSHV miRNAs directly target host lncRNAs

Of the 126 rescued lncRNAs identified based on transcriptional profiling, 98 contained seed sequence matches for at least one KSHV miRNA. Repeated sampling of 126 sequences from randomly generated DNA sequences, controlling for lncRNA length, revealed that the presence of KSHV miRNA seed matches in 98 out of 126 lncRNAs is statistically significant (p-value = 5.79 x 10^−8^, one-sided t-test). These data provide genetic evidence for miRNA-dependent deregulation of host lncRNAs during KSHV latency.

**Fig 3:**
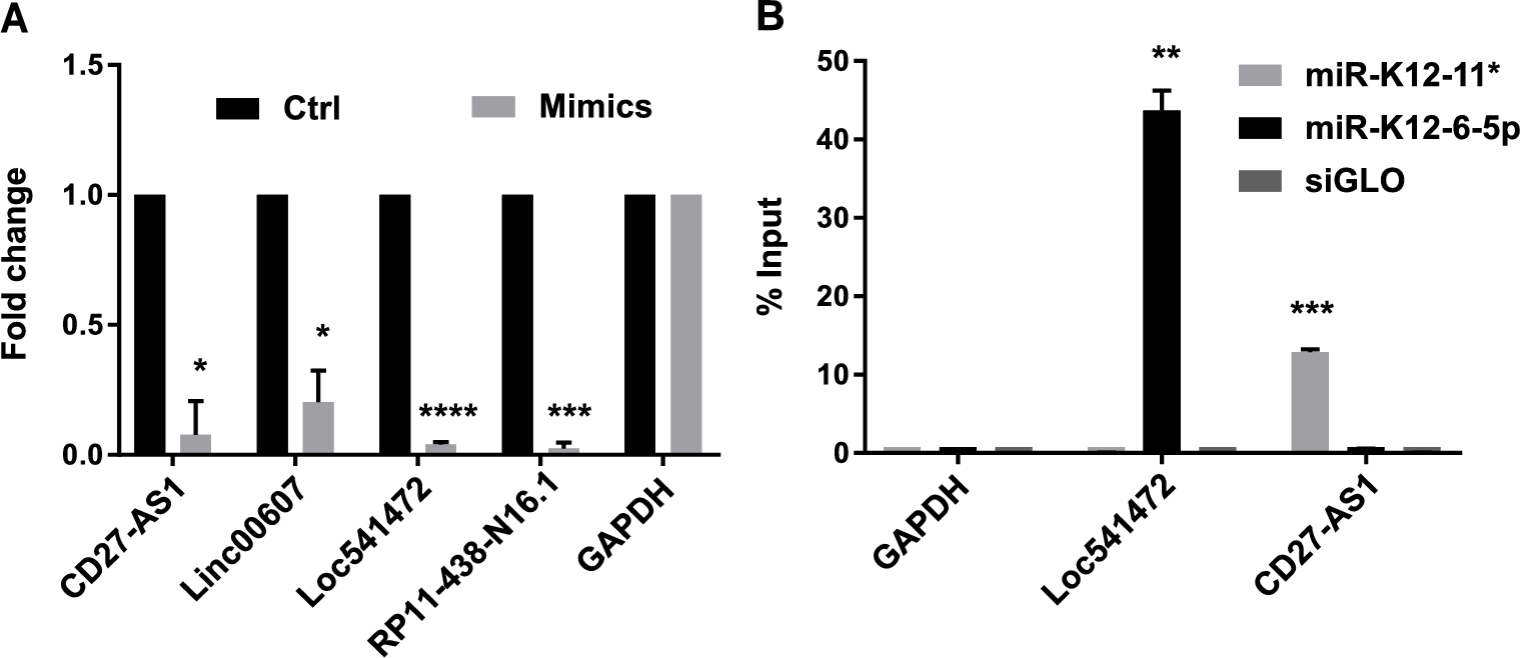
KSHV miRNAs directly bind to and downregulate host lncRNAs. **(A)** Uninfected TIVE cells were transfected with 5 nM final concentration of miRNA mimic pools (*Loc541472*: miR-K12-1, K12-6-5p; *CD27-AS1*: miR-K12-1*, K12-11*; *RP11-438-N16*.*1*: miR-K12-1*, K12-8*, K12-11*; *Linc00607*: miR-K12-2*, K12-11*). Relative expression levels of target lncRNAs were analyzed 48 h post-transfection using qRT-PCR. The bar graphs show the mean values ± SEM after normalization to GAPDH (n = 3). **(B)** Biotinylated miRNA mimics of miR-K12-6-5p and miR-K12-11* were transfected into uninfected TIVE-ExLTC cells (5 nM final concentration) and were pulled down 24 h later. Target lncRNAs were analyzed using qRT-PCR. siGLO pulldown was used a negative control. The bar graphs show the mean values ± SEM after normalization to input (n = 3). p-values: * < 0.01; ** < 0.005; *** < 0.0005; and **** <0.0001.

In order to validate that KSHV miRNAs can target host lncRNAs in the absence of KSHV infection, we chose four lncRNAs from the 98 containing seed sequences, and transfected pools of corresponding miRNA mimics into uninfected TIVE cells. The pools of mimics transfected were specific to the seed matches that those lncRNAs contained. Their respective mimic pools when compared to control mimic significantly knocked down all four lncRNAs tested, demonstrating that the viral miRNAs target lncRNAs in the absence of KSHV infection (**Fig 3A**).

The miRNA-dependent downregulation of lncRNAs could result from direct targeting of lncRNAs by miRNAs, or from an indirect secondary effect (e.g., through miRNA-mediated downregulation of transcription factors). To investigate direct interaction between KSHV miRNAs and lncRNAs, we performed miRNA pull-down experiments in TIVE-Ex-LTC cells. TIVE-Ex-LTC cells were derived from TIVE cells (see Materials and Methods), but grow much faster compared to TIVE cells. KSHV negative TIVE-Ex-LTC cells were transfected with biotinylated miRNA mimics for either miR-K12-6-5p, miR-K12-11* or siGLO (lacks biotin) and pull-down experiments were performed 24 h post-transfection. It is important to note that the mimics are dsRNAs that require loading into the RISC in order to bind their targets. Loc541472 has one binding site for miR-K12-6-5p but none for miR-K12-11*, and CD27-AS1 has one binding site for miR-K12-11* but none for miR-K12-6-5p. Biotinylated miR-K12-6-5p mimic pulled down 43.7% of Loc541472 and none of CD27-AS1, and miR-K12-11* mimic pulled down 12.9% of CD27-AS1, but no Loc541472, thus confirming direct miRNA-lncRNA interaction (**Fig 3B**). The fact that we identified putative lncRNA targets of viral miRNAs in PEL and endothelial cells by Ago HITS-CLIP and viral genetics, together with biochemical evidence for direct miRNA-lncRNA interaction, demonstrated that KSHV deregulates a subset of host lncRNAs in a miRNA-dependent fashion.

### Latent KSHV deregulates lncRNAs aberrantly expressed in cancer

To date a very small percentage of all lncRNAs are functionally annotated, making interpretation of lncRNA expression data challenging. As a starting point, we analyzed lncRNAs that were deregulated (upregulated, downregulated and rescued) in response to latent KSHV infection for known or proposed functions in disease processes. Comparison of our dataset to two public databases [28, 29] identified 54 lncRNAs that were previously shown to be aberrantly expressed in various human cancers (**S3 Table**). These include HOTTIP, DLEU2, HOTAIRM1, ANRIL, MEG3 and UCA1. Ten of the 54 lncRNAs are listed in **Table 2,** and include oncogenic and tumor suppressor lncRNAs. HOTTIP is upregulated in hepatocellular carcinoma, osteosarcoma, lung, prostate and other cancers [30]; DLEU2 is deleted in lymphocytic leukemia and epigenetically silenced in myeloid leukemia [31, 32]. Knockdown of HOTARM1 has been shown to promote proliferation in promyelocytic leukemia cells [33]. ANRIL is an oncogenic lncRNA that promotes proliferation in numerous cancers including basal cell carcinoma (BCC), glioma, prostate and ovarian cancers [34]. UCA1 is upregulated in multiple cancers, including bladder, endometrial and pancreatic cancer and acts as an oncogenic lncRNA [35]. Loss of MEG3 expression has been reported in a wide spectrum of malignancies ranging from gliomas to colon and liver cancers [36]. To understand the mechanisms by which cancer-related lncRNAs are deregulated by KSHV, and their contribution to pathogenesis, we chose to initially study UCA1, ANRIL and MEG3.

**Table 2:** Examples of oncogenic and tumor-suppressor lncRNAs deregulated by KSHV.

lncRNA	Function	Comparison group(s)	Ref.
ANRIL (CDKN2B-AS1)	Epigenetic silencing of tumor suppressor INK4B	KSHV vs. Mock: Down, Δcluster vs. Mock: Down	[34]
CRNDE	Upregulates mTOR pathway in gliomas, recently shown to code for a short nuclear peptide	KSHV vs. Mock: Down, Δcluster vs. KSHV: Up	[37]
DLEU2	Host gene for tumor suppressor miRNAs miR-15a and miR-16-1	KSHV vs. Mock: Up, Δcluster vs. Mock: Up	[31, 32]
HOTAIRM1	Modulates gene expression of cell adhesion molecules	KSHV vs. Mock: Up, Δcluster vs. Mock: Up	[33]
HOTTIP	Upregulates transcription of the antisense transcript, HOXA13	KSHV vs. Mock: Up, Δcluster vs. KSHV: Down	[30]
MEG3	Enhances p53 transcription and p53 responsive promoter transcriptions	KSHV vs. Mock: Up, Δcluster vs. KSHV: Up, Δcluster vs. Mock: Up	[36]
PLAC2 (TINCR)	Binds to Stau1 protein and regulates KLF2 mRNA in cells	KSHV vs. Mock: Up, Δcluster vs. Mock: Up	[38]
PTCSC3	Tumor suppressor lncRNA that acts by downregulating S100A4	KSHV vs. Mock: Down, Δcluster vs. Mock: Down	[39]
UCA1	Promotes cell cycle progression via PI3K-AKT pathway; also aids pRb1 and SET1A interplay	KSHV vs. Mock: Up, Δcluster vs. Mock: Up	[35]
ZEB1-AS1	Promotes EMT by upregulating ZEB1, MMP2, MMP9, N-cadherin, and Integrin-β1	KSHV vs. Mock: Down	[40]

### Viral miRNAs downregulate tumor suppressor lncRNA MEG3

**Fig 4:**
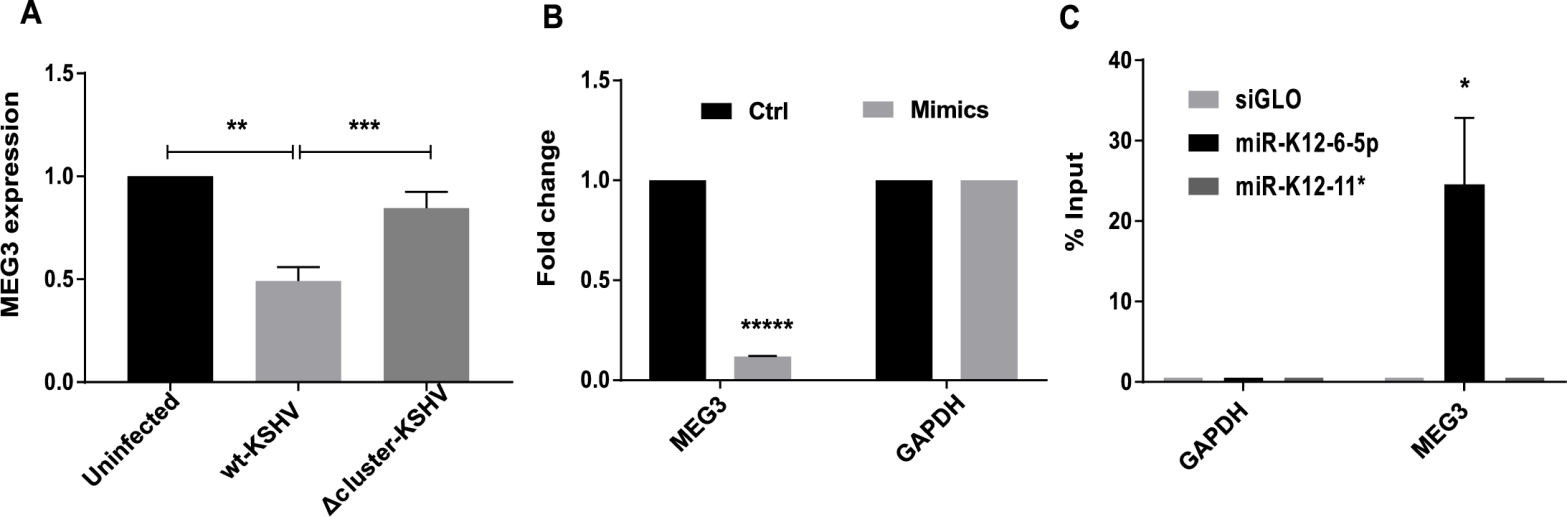
Tumor suppressor lncRNA MEG3 is targeted by KSHV miRNAs. All bar graphs show the mean values ± SEM after normalization to GAPDH (n = 3), unless specified otherwise. **(A)** MEG3 expression in Uninfected, wt-KSHV-infected and Δcluster-KSHV-infected cells measured by qRT-PCR. **(B)** Uninfected TIVE cells were transfected with 5 nM final concentration of miRNA mimic pool (miR-K12-5, K12-6-5p and K12-8*). Relative expression level of MEG3 was analyzed 48 h post-transfection using qRT-PCR. **(C)** Biotinylated miRNA mimic of miR-K12-6-5p was transfected into uninfected TIVE cells (5 nM final concentration) and was pulled down 24 h later. MEG3 expression was analyzed using qRT-PCR. siGLO pulldown was used as a negative control. The data were normalized to input. p-values: * < 0.05; ** < 0.01; *** < 0.005; **** < 0.0005; and ***** < 10^−4^.

MEG3 is a tumor suppressor lncRNA which is proposed to act by enhancing transcription from p53-dependent promoters [36]. Studies in HCT116 and U2OS cell lines have identified that MEG3 is a nuclear localized lncRNA [41], which was also confirmed in GM12878 cells by the GENCODE project [42]. According to the microarray data (**S2 Table**), MEG3 was slightly upregulated during latent KSHV infection. However, when validating MEG3 expression by qRT-PCR, it behaved in a rescued pattern, being suppressed in wt-KSHV infection and restored in Δcluster-KSHV-infected cells, suggesting regulation by KSHV miRNAs (**Fig 4A**). MEG3 contained seed sequence matches for miR-K12-3, K12-5, K12-6-5p, K12-8* and K12-9*. Uninfected TIVE cells were transfected with a pool of three KSHV miRNA mimics (miR-K12-5, K12-6-5p and K12-8*). MEG3 expression was reduced by almost 80% (**Fig 4B**). Furthermore, miRNA pull-down assays using biotinylated miR-K12-6-5p mimic pulled-down 24.5% of MEG3 (**Fig 4C**). miR-K12-11* mimic did not pull down MEG3 lncRNA. These data are consistent with viral miRNAs directly binding to and downregulating MEG3.

### Viral miRNAs and latency proteins both target ANRIL

**Fig 5:**
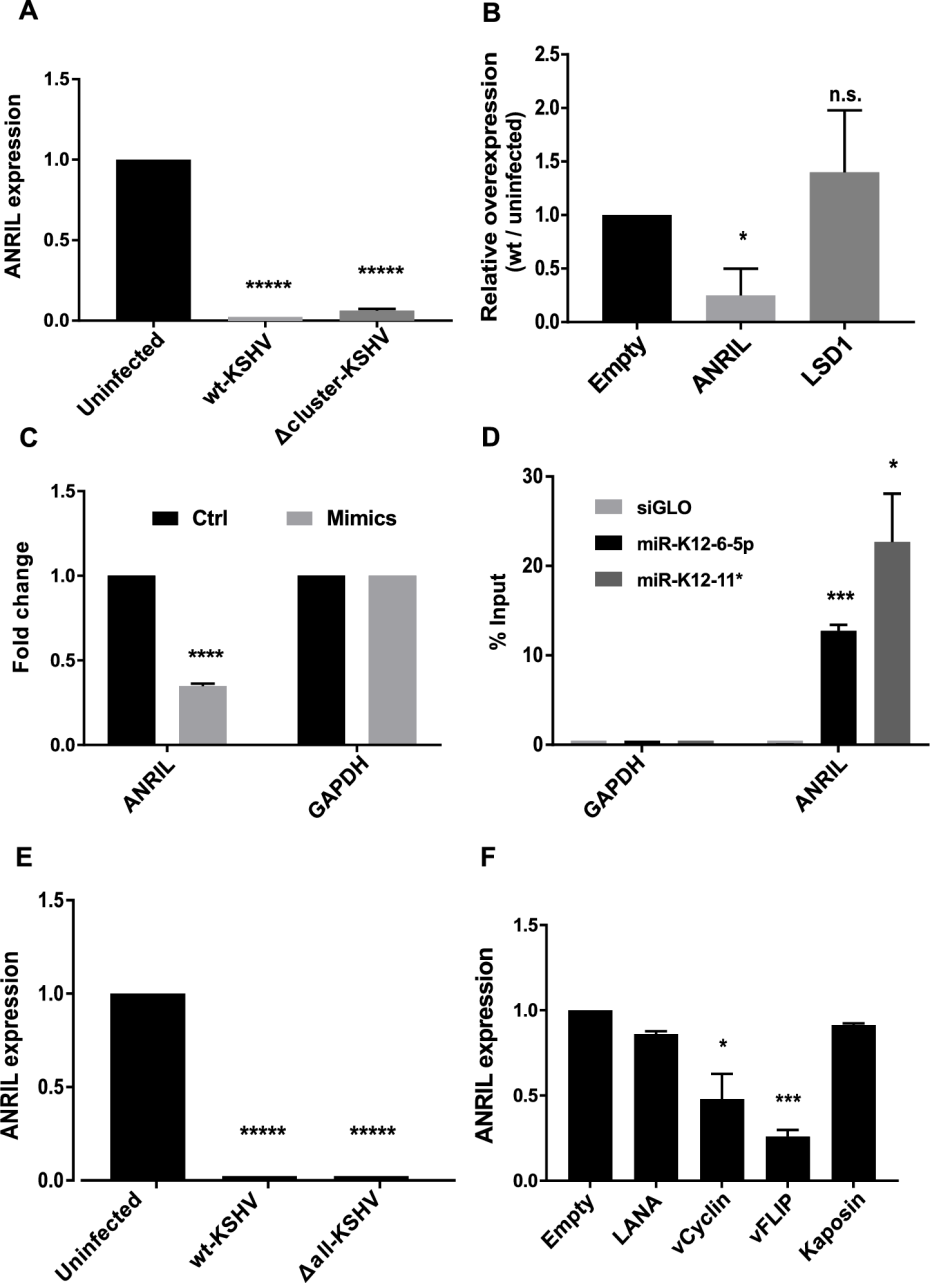
LncRNA ANRIL is targeted by both KSHV miRNAs and latency proteins. All bar graphs show the mean values ± SEM after normalization to GAPDH (n = 3), unless specified otherwise. **(A)** ANRIL expression in Uninfected, wt-KSHV-infected and Δcluster-KSHV-infected cells measured by qRT-PCR. **(B)** Uninfected and wt-KSHV-infected TIVE cells were transfected with pcDNA3.1-ANRIL and relative over-expression of ANRIL was measured using qRT-PCR. LSD-1 was used a control to verify comparable transfection efficiencies of uninfected and infected cells. Y-axis is calculated as the ratio of fold-overexpression observed in wt-KSHV infected cells to the fold-overexpression observed in uninfected cells. Overexpressions were normalized to any expression changes observed by transfecting empty vector, which is thus set at one. **(C)** Uninfected TIVE cells were transfected with 5 nM final concentration of miRNA mimic pool (miR-K12-1*, K12-6-5p, K12-2* and K12-11*). Relative expression level of ANRIL was analyzed 48 h post-transfection using qRT-PCR. (**D**) Biotinylated miRNA mimics of miR-K12-6-5p and miR-K12-11* were transfected into uninfected TIVE cells (5 nM final concentration) and were pulled down 24 h later. ANRIL expression was analyzed using qRT-PCR. siGLO pulldown was used as a negative control. The data were normalized to input. **(E)** ANRIL expression in Uninfected, wt-KSHV-infected and Δall-KSHV-infected cells measured by qRT-PCR (n = 2). **(F)** HeLa cells were transfected with latency gene(s) (LANA, vCyclin, vFLIP, Kaposin or vCyclin + Kaposin) expressed from pcDNA3.2 vector. ANRIL expression was analyzed 72 h post-transfection using qRT-PCR. p-values: * < 0.05; ** < 0.01; *** < 0.005; **** < 0.0005; ***** < 10^−4^ and n.s. = not significant.

ANRIL is a nuclear localized oncogenic lncRNA that drives proliferation by silencing the INK4 tumor suppressor gene by recruiting PRC2 complexes [34]. The fact that ANRIL was downregulated in KSHV-infected cells from the microarray data suggested that ANRIL does not have a direct role in proliferation; however, ANRIL has recently also been implicated in innate immune responses, albeit in the context of bacterial infection [43]. Analysis of ANRIL expression by qRT-PCR showed a very strong 100-fold downregulation in wt-KSHV-infected cells, and a slightly reduced inhibition in the Δcluster-KSHV-infected TIVE cells (**Fig 5A**). Such strong repression is not typical of miRNAs, however, the cDNA of ANRIL had a total of 17 6-mer seed matches for 12 of 25 mature KSHV miRNAs. To investigate whether the large number of KSHV miRNA seed sequence matches in ANRIL are targeted by KSHV miRNAs, we ectopically overexpressed the shortest isoform (transcript variant 12) of ANRIL from a CMV promoter-driven vector in wt-KSHV-infected and uninfected TIVE-Ex-LTC cells. Since TIVE cells are highly resistant to plasmid transfection, we used TIVE-Ex-LTC cells for this experiment. As shown in **Fig 5B**, the ANRIL expression levels achieved in wt-KSHV-infected cells were 80% less compared to uninfected cells. We note that this expression difference was not due to differences in transfection efficiencies, since a control gene (LSD-1), expressed from the same vector, was expressed at similar levels in both cell lines (**Fig 5B**). Hence, the reduced ANRIL expression levels in infected cells compared to control cells strongly suggested post-transcriptional miRNA-dependent regulation of ANRIL. To test this, we transfected a pool of four miRNA mimics (miR-K12-1*, K12-6-5p, K12-2* and K12-11*) which led to a strong knock-down of ANRIL expression in uninfected TIVE cells compared to the control mimic (**Fig 5C**). Additionally, pull-down experiments in TIVE cells using biotinylated miR-K12-6-5p and miR-K12-11* mimics, for which ANRIL contains two seed matches each, significantly pulled-down 12.7% and 22.7% of ANRIL transcripts, respectively (**Fig 5D**).

Together these data show that ANRIL is targeted by multiple viral miRNAs. Since ANRIL also contained miRNA seed sequence matches for miR-K12-10 and K12-12, which are still present in Δcluster-KSHV (**Fig 1A**), we wanted to test ANRIL expression in the absence of all viral miRNAs. To this end we analyzed ANRIL expression in TIVE cells by infecting with a virus lacking all 12 miRNA genes (Δall-KSHV). Surprisingly we did not observe significantly altered ANRIL expression compared to wt-KSHV-infected cells (**Fig 5E**). These data suggested that ANRIL may also be negatively regulated by latency associated proteins. To directly address this question we ectopically expressed the major latency associated proteins of KSHV (LANA, vCyclin, vFLIP and Kaposin) and monitored ANRIL expression by qRT-PCR. Since TIVE-Ex-LTC cells do not express detectable levels of ANRIL, this experiment was performed in HeLa cells, which are known to robustly express ANRIL [44]. vFLIP and vCyclin downregulated ANRIL expression by almost 75% and 53%, respectively (**Fig 5F**). LANA and Kaposin did not have significant effects. The observation that ANRIL is negatively regulated by both miRNAs and latency associated proteins is in congruence with other host genes that are targeted by multiple viral mechanisms [45].

### miRNA-independent deregulation of host lncRNA UCA1 promotes proliferation and migration

**Fig 6:**
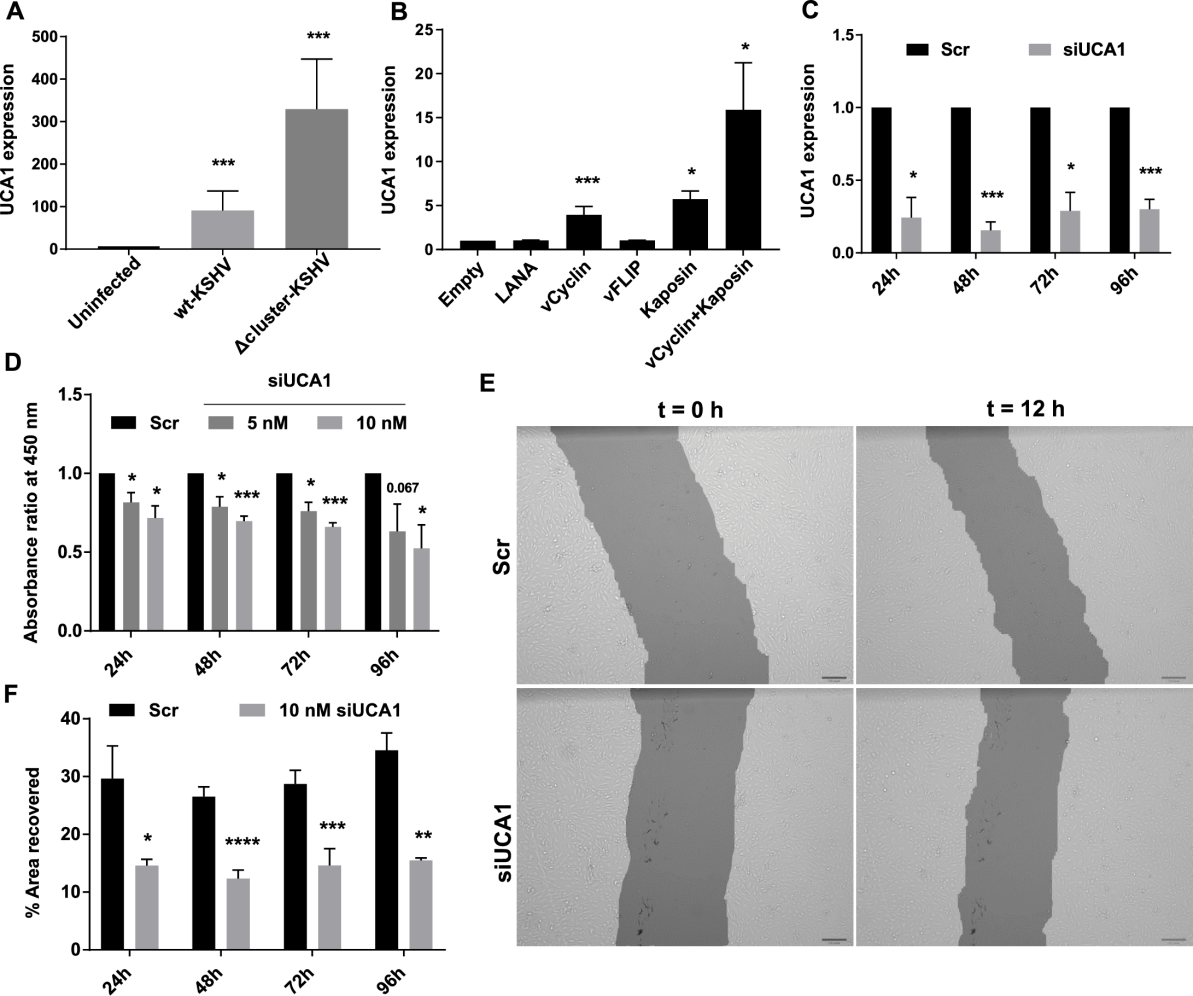
UCA1 is upregulated by KSHV in a miRNA-independent manner. **(A)** UCA1 expression in Uninfected, wt-KSHV-infected and Δcluster-KSHV-infected cells measured by qRT-PCR. The bar graphs show the mean values ± SEM after normalization to GAPDH (n = 6). **(B)** Uninfected TIVE cells were transfected with latency gene(s) (LANA, vCyclin, vFLIP, Kaposin or vCyclin + Kaposin) expressed from pcDNA3.2 vector. UCA1 expression was analyzed 72 h post-transfection using qRT-PCR. The bar graphs show the mean values ± SEM after normalization to GAPDH (n = 6 for vCyclin, n = 3 for others). **(C)** wt-KSHV-infected TIVE cells were transfected with 10 nM concentration of siUCA1 or Scr control. At 24, 48, 72 and 96 h, UCA1 expression was analyzed using qRT-PCR. The bar graphs show the mean values ± SEM after normalization to GAPDH (n = 3). **(D)** wt-KSHV-infected TIVE cells were transfected with 5 nM or 10 nM concentration of siUCA1 or Scr control. At 24, 48, 72 and 96 h, the samples were subject to MTS assay and absorption was measured at 495 nm wavelength. The bar graphs show the relative absorbance ± SEM (n = 3). **(E and F)** wt-KSHV-infected TIVE cells were transfected with 10 nM concentration of siUCA1 or Scr control. At 24, 48, 72 and 96 h, the samples were subject to scratch assay. Plates were imaged at 0 and 12 h and the images were processed using T-Scratch. **(E)** Representative images of the wound healing assay, scale bar = 100 pixels. **(F)** The bar graphs show the percentage of scratch area recovered ± SEM (n = 3). p-values: * < 0.05; ** < 0.01; *** < 0.005; and **** < 0.0005.

Urothelial Cancer Associated 1 (UCA1) is a lncRNA which was identified as highly upregulated in bladder cancer and has since been implicated in other cancers like colorectal, ovarian and renal carcinomas [35]. UCA1 is partially localized in both the nucleus and the cytoplasm and plays distinct roles in different sub-cellular compartments [46, 47]. Recently, it was shown that UCA1 transcription is induced by HIF-1α, to enhance hypoxic proliferation, migration and invasion of bladder cancer cells [35]. UCA1 was upregulated by approximately 90-fold during wtKSHV infection and approx. 300-fold during Δcluster-KSHV infection (**Fig 6A**). Since UCA1 was upregulated under both infection conditions and its cDNA sequence contained no seed matches for any KSHV miRNAs, UCA1 is presumably not regulated by a miRNA-dependent mechanism.

To determine which of the four major latency-associated proteins (LANA, vCyclin, vFLIP and Kaposin) upregulates UCA1, we transfected TIVE-Ex-LTC cells with expression vectors either alone or in combination. Ectopic expression of vCyclin and Kaposin led to a 3.9 and 5.7-fold upregulation of UCA1 as monitored by qRT-PCR, respectively. Furthermore, co-transfection of vCyclin and Kaposin increased UCA1 to almost 15-fold compared to empty vector suggesting synergy (**Fig 6B**). LANA and vFLIP had no effect. The fact that the upregulation observed in transfected cells is much less than in the context of infection could be a consequence of either an altered stoichiometry or absolute expression levels of latency proteins, or mean that other viral genes might contribute to UCA1 upregulation.

To address whether UCA1 directly contributes to KS-associated phenotypes, we knocked-down UCA1 expression using siRNAs in KSHV-infected TIVE cells. At 24, 48, 72 and 96 h post-transfection we observed 60–85% knockdown of UCA1 expression (**Fig 6C**). First, we assayed for proliferation using the MTS assay. We measured proliferation at 24, 48, 72 and 96 h post-transfection and observed a statistically significant and dose-dependent decrease in proliferation of cells treated with siUCA1 as compared to scrambled control (Scr). Upon treatment with 10 nM siUCA1, the proliferation rate dropped to 72% by day 1 and then progressively to 52% by day 4 (**Fig 6D**). We observed a similar decrease in proliferation of uninfected TIVE cells transfected with siUCA1 (**S2A Fig**) suggesting that UCA1 contributes to endothelial cell proliferation in general. To test whether latent KSHV upregulates UCA1 in all infected cells, we measured UCA1 expression levels in uninfected and KSHV-infected iSLK cells. UCA1 was upregulated by almost 5-fold in KSHV-infected iSLK cells (**S3A Fig**). Knockdown of UCA1 in uninfected and KSHV-infected iSLK cells led to a mild reduction in proliferation of these cells (**S3B and S3C Fig**). The magnitude of effect observed in iSLK cells was much lower than that in TIVE cells, presumably because iSLK cells are transformed and unlike TIVE cells form tumors in nude mice [18]. Next, we assayed the effect of UCA1 knockdown on migration of KSHV-infected TIVE cells. The migration assay (wound healing) involves introduction of a scratch in a monolayer of cells and measuring the percentage of the clear area that gets covered by migration at 12 hours post introduction of the scratch under serum-free conditions (**Fig 6E**). siUCA1-treated cells were consistently slower in migration from day 1 through day 4, as they recovered only between 12–15% of the scratch area, while Scr-treated cells recovered between 26–35% of the area (**Fig 6F**). A similar reduction in migration was observed on days 1 and 2 when UCA1 was knocked down in uninfected TIVE cells, however, no difference was evident after day 3 (**S2B Fig**). This suggests that high UCA1 levels in KSHV-infected endothelial cells contribute to increased migration of these cells. These data demonstrate that the induction of UCA1 by the KSHV latency-associated proteins Kaposin and vCyclin promotes proliferation and migration, and likely contributes to KSHV pathogenesis and tumorigenesis.

## Discussion

Here we show that latent KSHV infection significantly alters the lncRNA expression profile of endothelial cells. Deregulation of lncRNAs has implications in diseases such as diabetes, neurological disorders, viral infections and cancer [48, 49]. Our study establishes that KSHV employs its latency proteins and miRNAs, either alone or in combination, to target specific lncRNAs and potentially contribute to sarcomagenesis.

Post-transcriptional regulation of lncRNA expression by miRNAs is a newly described phenomenon. Yoon et al showed let-7 loaded RISCs targeted lincRNA-p21 in a HuR-dependent manner in cervical carcinoma cells, eventually destabilizing and degrading lincRNA-p21 [50]. In bladder cancer, UCA1 and miR-1 expressions were inversely correlated, and overexpression of miR-1 phenocopied the knockdown of UCA1 [51]. Further, MALAT-1, a nuclear lncRNA, was reported to be targeted by miR-9 in an Ago-2-dependent manner in the nuclei of Hodgkin’s lymphoma and glioblastoma cell lines [15]. We identified 126 lncRNAs as potential targets of viral miRNAs in endothelial cells, and we verified direct miRNA/lncRNA interactions by pulldown experiments with biotinylated KSHV miRNA mimics targeting Loc541472, CD27-AS1, ANRIL and MEG3. Results from the Ago HITS-CLIP experiment further suggest that this regulation proceeds in an Ago and hence RISC-dependent manner. As per our current understanding, RISC-mediated silencing of mRNAs proceeds via translation repression and induction of mRNA turnover [52, 53]. RNA destabilization followed by degradation is perhaps the mechanism relevant to silencing of lncRNAs. However, the details of the mechanism, especially for lncRNAs lacking a cap and/or a poly-A tail, remain to be uncovered.

An alternative and not mutually exclusive mechanism that involves direct engagement of miRNAs and lncRNAs is miRNA sponging by lncRNAs [54]. LincRNA-RoR sponges miR-145-5p thereby increasing the expression of pluripotent stem cell factors Oct4, Nanog and Sox2 [55]. The Steitz lab showed that lncRNAs encoded by Herpesvirus Saimiri, called HSURs, sequester host miRNAs in infected T-lymphocytes [16]. It is plausible that some host lncRNAs could sponge KSHV miRNAs, thereby derepressing downstream targets instead of being targeted by miRNAs themselves.

We demonstrated that viral latency proteins vCyclin and Kaposin synergistically upregulate UCA1 while vFLIP and vCyclin downregulate ANRIL. Thus, aside from miRNAs, the latency proteins play a pronounced role in perturbing lncRNA expression. This is not surprising given we identified 858 differentially expressed lncRNAs during wt-KSHV infection and only 126 were potential miRNA targets. vCyclin, an ortholog of cellular Cyclin D, upregulates expression of cell cycle regulatory genes [56]. Moreover, Kaposin stabilizes cytokine mRNAs thereby increases their turnover time [57]. vCyclin and Kaposin may act cooperatively by augmenting transcription and simultaneously preventing turnover of UCA1. We also showed that ectopically expressed vFLIP strongly downregulates ANRIL. STAT1-mediated activation of the ANRIL locus in vascular endothelial cells has been reported based on GWAS studies [58]. Studies using a mutant virus that lacks vFLIP in HUVEC cells showed activation of STAT1 in a NF-κB-dependent manner, suggesting that vFLIP probably inhibits STAT1 to downregulate ANRIL expression [59]. A recent study in endothelial cells demonstrated that ANRIL expression is induced by pro-inflammatory molecules, especially NF-κB and TNF-α, and silencing of ANRIL expression led to a reduction in IL6/IL8 response [60]. This further underlines the role of ANRIL in immunity and supports the notion that KSHV may downregulate ANRIL to evade innate immune responses.

KSHV drives latently infected cells towards proliferation by a variety of mechanisms such as encoding orthologs for cell cycle proteins like vCyclin, or interfering with the p53 pathway through LANA [61], encoding miR-K12-11, an ortholog of oncomir-155 [62], and the induction of the oncogenic host miRNA cluster miR-17/92 [45]. Here we demonstrate that KSHV also upregulates UCA1 to drive proliferation and migration in endothelial cells. UCA1 has also been shown to promote the Warburg effect [63], an effect that has been shown to be required for maintenance of latent KSHV in endothelial cells [64]. We found that 53 additional lncRNAs previously shown to be aberrantly expressed in various malignancies are deregulated by KSHV, suggesting that UCA1 exemplifies how KSHV could similarly exploit lncRNAs that contribute to phenotypes such as proliferation and migration in the context of tumorigenesis. Given that the majority of lncRNAs we catalogued in this study remain uncharacterized, the repertoire of cancer-relevant lncRNAs regulated by KSHV may be much larger. Although cancer is the pathological consequence of KSHV infection, KSHV could target lncRNAs of biological significance in other cellular processes, for example, lncRNAs involved in inflammation and innate immunity [9]. KSHV continually evades the innate immune response using several approaches, like suppressing TGF-β signaling [45], activation of NF-κB response genes [65] and encoding trace amounts of v-IL6, a truncated version of human IL-6, during latent infection [66]. Loc541472, which we show here is targeted directly by KSHV miRNAs, is antisense to the hIL-6 promoter, suggesting that targeting of this lncRNA contributes to regulation of IL-6 expression. Indeed, preliminary experiments suggest a correlation between Loc541472 and hIL-6 expression and mechanistic studies are currently ongoing.

We identified a novel paradigm by which KSHV, an oncogenic herpesvirus, regulates cellular gene expression by targeting host lncRNAs with viral miRNAs and latency proteins. Studying lncRNAs deregulated by KSHV may yield novel mechanisms by which viruses evade the host immune response and in the case of EBV and KSHV contribute to tumorigenesis, as exemplified by our data on UCA1 which modulates migration and proliferation. Finally, studies on aberrantly expressed lncRNAs in KSHV-infected cancer cells may aid the functional characterization of cellular lncRNAs and at the same time identify novel virus-specific therapeutic targets for KS.

## Material and methods

### Virus and plasmid constructs

The viruses used in this study, wt-KSHV, Δcluster-KSHV and Δall-KSHV, have the viral genome cloned into a Bac-16 backbone, as described in Brulois et al. [19] and Jain et al. [20]. Transcript variant 12 (RefSeq ID: NR_047542.1) of ANRIL was expressed from a pcDNA3.1 vector [67]. LANA, vCyclin, vFLIP and Kaposin were expressed from pcDNA3.2 vectors [68].

### Cell culture

Telomerase immortalized vein endothelial cells (TIVE) and long-term cultured KSHV infected cells (TIVE-LTC) were generated by immortalizing passage 2 HUVEC cells (kindly provided by Dr. Keith McCrae, Case Western Reserve University) in our laboratory as described [18]. All uninfected and infected TIVE cells were grown in complete Medium-199 (1% Pen-Strep, 20% FBS), supplemented with Endothelial cell growth supplement (Sigma). TIVE-Ex-LTC cells were obtained by culturing TIVE-LTC cells as single cell dilutions without antibiotic selection, and have lost all copies of viral episomes. TIVE-Ex-LTC cells grow faster and are more transfectable compared to TIVE cells. All uninfected and infected TIVE-Ex-LTC cells were grown in complete DMEM (1% Pen-Strep, 10% FBS). Latently infected TIVE and TIVE-Ex-LTC cells were maintained under hygromycin (10 μg/mL) to prevent episome loss. Body-cavity-based lymphoma (BCBL-1) cell line was derived from KSHV positive primary effusion lymphoma (PEL) and was kindly provided by Dr. Don Ganem at UCSF [69]. BCBL-1 cells were grown in complete RPMI (2% Pen-Strep, 10% FBS). HeLa cells and iSLK cells were grown in complete DMEM (1% Pen-Strep, 10% FBS).

### Bioinformatics analysis

#### Reanalysis of CLIP data

The BED files generated as a part of the analysis of Ago HITS-CLIP data from PEL cells [3] were compared with GENCODE V19 [70] to obtain a comprehensive list of putative lncRNA targets. These lncRNAs were compared with published tables available from EBV PAR-CLIP [17]. *Statistical test for enrichment of KSHV miRNA seed matches in lncRNAs*: R version 3.3.0 was used for this analysis. 100,000 random DNA sequences of length 1189 nt were generated. This number was obtained by calculating the mean length of the 126 rescued lncRNAs. KSHV miRNA seed matches were counted using repeated sampling (10,000 times) of 126 random DNA sequences. One-sided t-test was performed to compare the average number of seed matches in random sequences to that of rescued lncRNAs.

### Fractionation of PEL cells

The method for isolating nuclear and cytoplasmic fractions was adapted from [71]. Briefly, 1 x 10^7^ BCBL-1 cells were pelleted and washed twice with ice cold PBS. Cells were resuspended smoothly by gentle pipetting in Sucrose buffer I (SB-I: 0.32 M Sucrose, 3 mM CaCl_2_, 2 mM Mg(Ac)_2_, 0.1 mM EDTA, 10 mM Tris-HCl (pH 8), 1 mM DTT, 0.5 mM PMSF and 0.5% NP-40) using 100 μL buffer per 1 x 10^7^ cells. Lysis was at room temperature for 60–90 s. The nuclei were pelleted at 800 x *g*, 4 ˚C for 5 min and the supernatant (cytoplasmic fraction) was frozen immediately and stored at -80C. The pellet was resuspended smoothly by gentle pipetting in 50 μL of SB-I and allowed to sit for 30 s at RT. The nuclei were pelleted again at 800 x *g*, 4 ˚C for 5 minutes. The supernatant was discarded and the pellet (now whiter) was washed twice in 1 mL ice cold PBS. The resuspension was smooth and easy indicating no nuclear rupture. 10 μL of the 1 mL suspension from the second wash was trypan blue stained and checked by microscopy to verify the purity and integrity of the isolated nuclei. The nuclear fraction was frozen immediately and stored at -80 ˚C.

### Immunofluorescence assays

TIVE cells were grown overnight on coverslips at a dilution of 1 x 10^4^ cells per well in a 6-well plate. Nuclei isolated from PEL cells were prepared as described [72], and fixed with a 1:1 ratio of methanol and acetone for 10 min in a humid chamber at 4 ˚C. The samples were blocked in PBS with 3% BSA for 1 h at room temperature, and then incubated overnight at 4 ˚C with either primary anti-Ago2 antibody or blocking solution (control). After washing, the samples were incubated with Alexa-468 anti-rat secondary antibody for 1 hour at room temperature. The slides were then stored at -20 ˚C and imaged using a LEICA TCS SP2 AOBS Spectral Confocal microscope. The images were analyzed and figures were generated using the freeware Vaa3D [73]. The movie was generated using Volocity® 6.3.

### Western blots

SDS-PAGE and Western blotting were performed using whole cell lysates, or cytoplasmic or nuclear fractions prepared from 100,000 cells/well. The following antibodies were used to probe the membrane: Ago2 (11A9, [74]), β-Tubulin (Millipore, CP06-100UG), Sm antigen (Dr. Joan Steitz’s lab, Yale University), Lamin A/C (Active Motif, 39288), Calnexin (ENZO Lifesciences, ADI-SPA-865-D).

### RNA isolation and microarray analysis

Total RNA was isolated with RNA-Bee (Tel-Test Inc.) using the protocol provided by the manufacturer. Total RNA (5–10 μg) was treated with DNase I (NEB) according to the manufacturer’s instructions and ethanol precipitated overnight. Genome-wide lncRNA microarray analysis was performed with ArrayStar using Human LncRNA Array v3.0 (8 x 60K, Arraystar). A fold change cut-off of 2.0 was applied to filter lncRNAs into different categories (upregulated, downregulated and rescued) for further analyses. Three technical replicates for each of the three samples were analyzed.

### qRT-PCR of miRNAs

Total RNA preparations from PEL cell fractions were reverse transcribed using the TaqMan MicroRNA Reverse Transcription Kit (ThermoScientific). Stem-loop qPCR was performed using the TaqMan Gene Expression Master Mix and appropriate miRNA assays from Applied Biosystems.

### qRT-PCR of mRNA or lncRNAs

Total RNA (2 μg) was reverse transcribed using SuperScript III (Life Technologies) using random hexamers according to the manufacturer’s instructions. cDNA corresponding to 50–100 ng RNA was used per 10 μL of qPCR reaction. Instruments used for real-time PCR included ABI StepOne Plus (Applied Biosystems) and LightCycler96 (Roche). qPCR primer sequences are listed in **S4 Table**.

### miRNA mimic transfections

TIVE cells were seeded in 48-well plates (50,000 cells/well) and transfected with pools of miRNA mimics (in equimolar ratios and a final concentration of 5 nM) purchased from Qiagen. At 48 h post transfection, the lncRNA expression levels were measured using the Power SYBR Green Cells-to-CT Kit (ThermoFisher). In the cases of ANRIL and MEG3, 10 cm plates were seeded to 70% confluency and qRT-PCR analysis was performed using the conventional approach described above.

### Biotinylated miRNA pulldown

Biotinylated miRNA mimics (miR-K12-6-5p and miR-K12-11*) were purchased from Exiqon. Pulldown was performed from TIVE and TIVE-Ex-LTC cells according to the previously published protocol [75] with minor changes. Each replicate started with 6x10^6^ cells for TIVE-Ex-LTCs (instead of 4x10^6^) and 8x10^6^ cells for TIVE cells. Input RNAs saved for analysis were 5% and 20% for TIVE-Ex-LTC and TIVE cells, respectively.

### Ectopic expression of latency genes from plasmids

TIVE-Ex-LTC cells were reverse transfected in 6-well plates (300,000 cells/ well) with 2 μg of plasmid DNA using FuGENE HD according to the manufacturer’s protocol. HeLa cells were seeded in 6-well plates (150,000 cells/ well) and were transfected 24 h later with 2 μg plasmid DNA using Lipofectamine 3000 according to the manufacturer’s protocol. DMEM (10% FBS) was used for transfection of both cell types. Comparable transfection efficiencies were ensured by co-transfection of pmaxGFP. Total RNA was harvested from transfected cells at 72 h post-transfection.

### siRNA knockdown

wtKSHV-infected or uninfected TIVE cells were plated in 96-well plates (20,000 cells/well for MTS assay) and 48-well plates (250,000 cells/well for wound healing assay). Uninfected and wtKSHV-infected iSLK cells were plated in 96-well plates (8000 cells/well for MTS assay). siRNAs (5nM or 10 nM) against UCA1 (Qiagen) were transfected using Lipofectamine RNAiMAX reagent (ThermoFisher) according to the manufacturer’s protocol. ON-TARGETplus Non-targeting Control siRNA (Dharmacon) was used as the scrambled negative control. At 4 h post-transfection, the serum free medium was replaced by complete Medium-199 (TIVE) or DMEM (iSLK). Comparable transfection efficiencies were ensured by co-transfection of siGLO (Dharmacon).

### Cell proliferation and migration assays

#### MTS assay

At 24, 48, 72 and 96 h post-transfection of siRNAs, the MTS assay was performed using the CellTiter 96 AQueous Non-Radioactive Cell Proliferation Assay kit (Promega) according to the manufacturer’s instructions. The absorbance of the samples was measured at 490 nm. *Wound-healing assay*: At 24, 48, 72 and 96 h post-transfection with siRNAs, the confluent wells were scratched using a 200 μL pipette tip along the diameter of the well. Images of the scratch were recorded at 0 and 12 h, and analyzed using the freeware Tscratch [76].

### Statistics

Statistical analyses on experimental measurements were done using two-tailed student’s t-test assuming unequal variances for all experiments reported.

### Data availability

Raw data files from the microarray experiment were deposited to the Gene Expression Omnibus under the accession number GSE89114.

## Supporting information

S1 Fig Validation of microarray results. All bar graphs show the mean values ± SEM after normalization to GAPDH (n = 2), unless specified otherwise. Expression levels of two downregulated lncRNAs, two upregulated lncRNAs, and three rescued lncRNAs were measured by qRT-PCR in uninfected, wt-KSHV-infected and Δcluster-KSHV-infected TIVE cells. In addition, data for ANRIL in the downregulated category and UCA1 in the upregulated category are shown in Fig 5A and Fig 6A, respectively.
(TIF)

S2 Fig Knockdown of UCA1 in uninfected TIVE cells. **(A)** Uninfected TIVE cells were transfected with 5 nM or 10 nM concentration of siUCA1 or Scr control. At 24, 48, 72 and 96 h, the samples were subject to MTS assay and absorption was measured at 495 nm wavelength. The bar graphs show the relative absorbance ± SEM (n = 3). **(B)** Uninfected TIVE cells were transfected with 10 nM concentration of siUCA1 or Scr control. At 24, 48, 72 and 96 h, the samples were subject to scratch assay. Plates were imaged at 0 and 12 h and the images were processed using T-Scratch. The bar graphs show the percentage of scratch area recovered ± SEM (n = 3). For 96 h time-point, those data points where the scratch area was completely recovered were omitted. p-values: * < 0.05; ** < 0.005; and *** < 0.0005.
(TIF)

S3 Fig Knockdown of UCA1 in uninfected and wt-KSHV-infected iSLK cells. **(A)** UCA1 expression in uninfected and wt-KSHV-infected iSLK cells measured by qRT-PCR. The bar graphs show the mean values ± SEM after normalization to GAPDH (n = 2). **(B)** wt-KSHV-infected iSLK cells were transfected with 5 nM or 10 nM concentration of siUCA1 or Scr control. At 24, 48, 72 and 96 h, the samples were subject to MTS assay and absorption was measured at 495 nm wavelength. The bar graphs show the relative absorbance ± SEM (n = 3). **(C)** Uninfected iSLK cells were transfected with 5 nM or 10 nM concentration of siUCA1 or Scr control. At 24, 48, 72 and 96 h, the samples were subject to MTS assay and absorption was measured at 495 nm wavelength. The bar graphs show the relative absorbance ± SEM (n = 3). p-values: * < 0.05; ** < 0.005.
(TIF)

S1 Movie Immunofluorescence of Ago2 localization in wt-KSHV infected TIVE cells. (MP4)

S1 Table List of lncRNAs that were identified in both Ago HITS-CLIP of PEL cells (KSHV infected) and Ago PAR-CLIP of LCL cells (EBV infected). (XLSX)

S2 Table Transcriptional profiling data. (XLSX)

S3 Table List of deregulated lncRNAs identified from microarray analysis and the cancer type where their aberrant expression has been reported. (XLSX)

S4 Table qRT-PCR primers and biotinylated miRNA mimic sequences. (PDF)
