# Supplementary figures and images for: microRNA dependent and independent deregulation of long non-coding RNAs by an oncogenic herpesvirus
Source: PLoS Pathog. 2017 Jul 17;13(7):e1006508. doi: 10.1371/journal.ppat.1006508 (PMC5531683; doi:10.1371/journal.ppat.1006508)

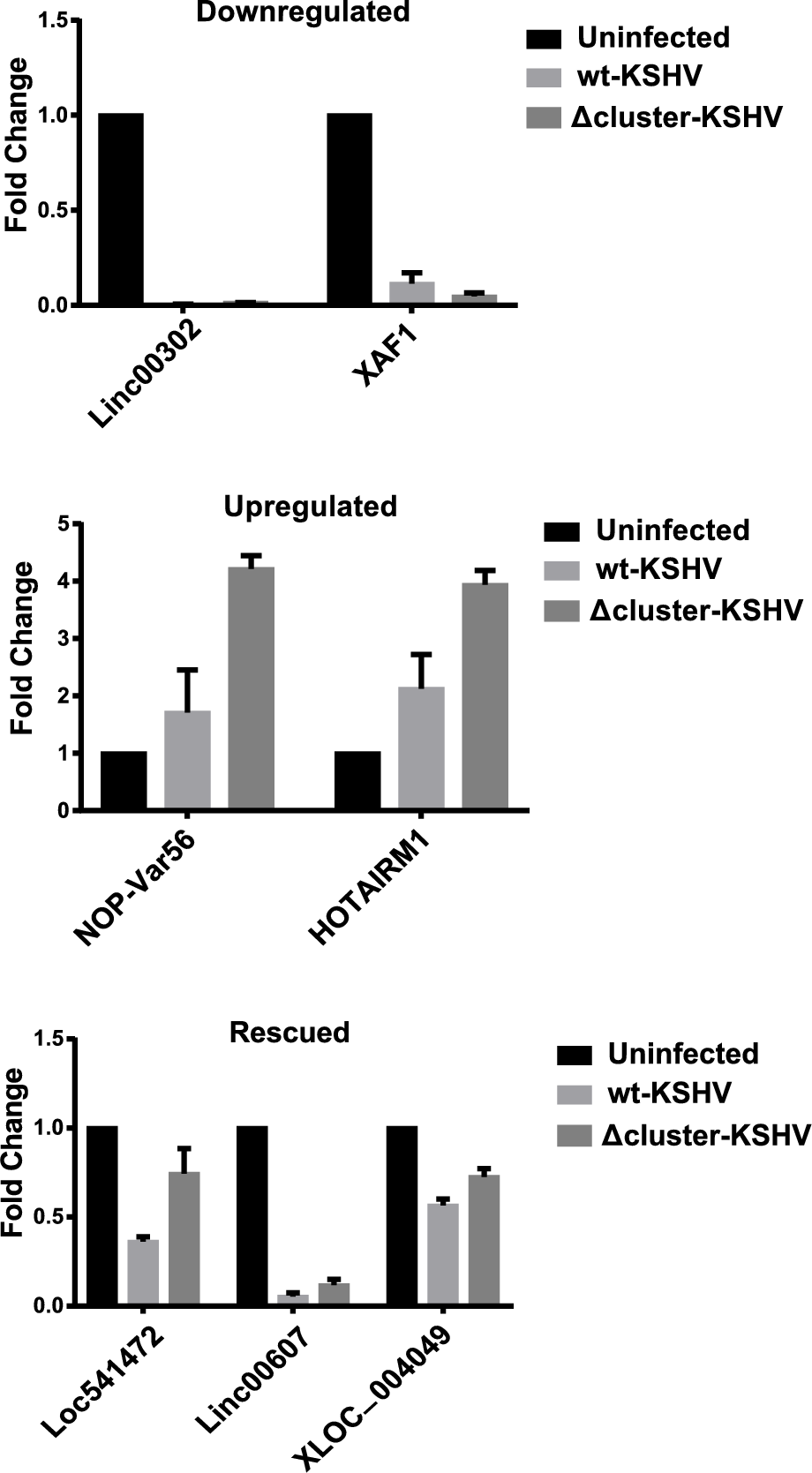

Supplement: S1 Fig — All bar graphs show the mean values ± SEM after normalization to GAPDH (n = 2), unless specified otherwise. Expression levels of two downregulated lncRNAs, two upregulated lncRNAs, and three rescued lncRNAs were measured by qRT-PCR in uninfected, wt-KSHV-infected and Δcluster-KSHV-infected TIVE cells. In addition, data for ANRIL in the downregulated category and UCA1 in the upregulated category are shown in Fig 5A and Fig 6A, respectively. (TIF) [file ppat.1006508.s001.tif]

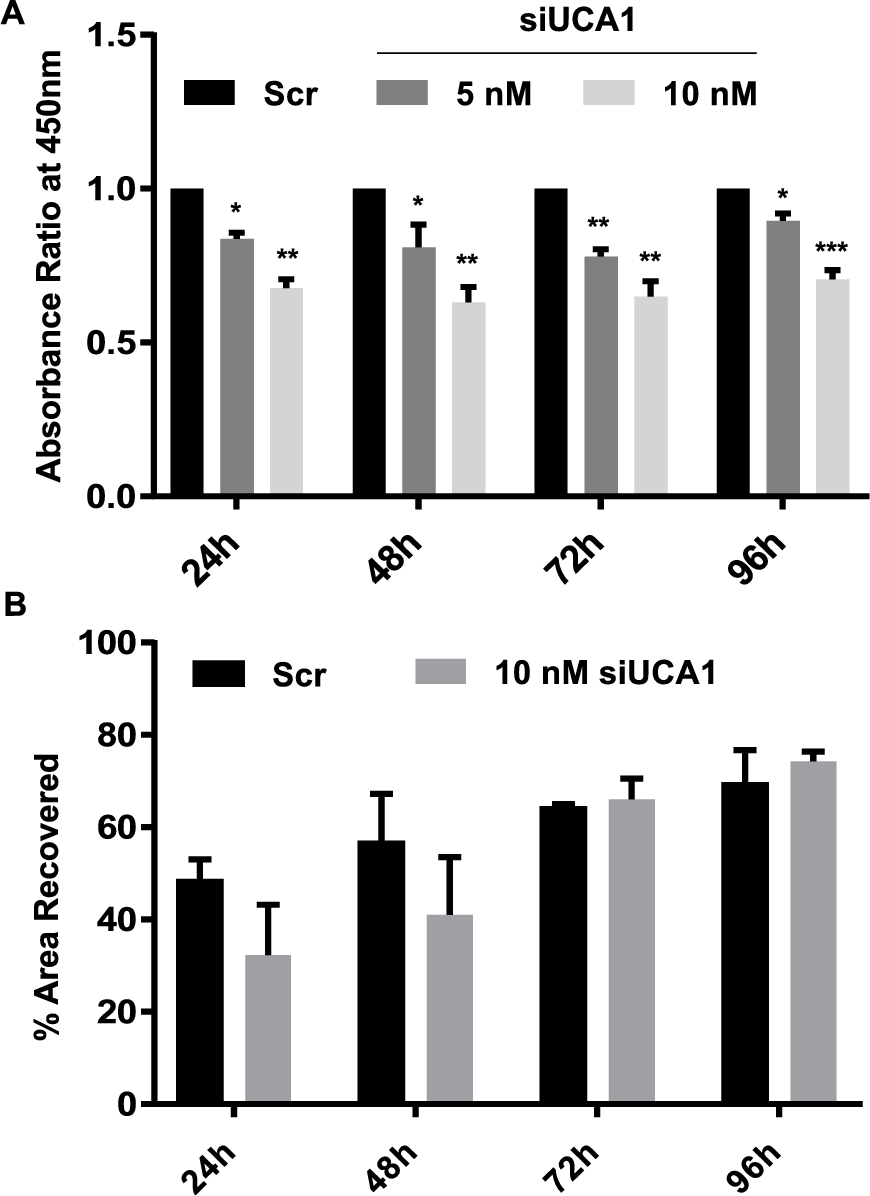

Supplement: S2 Fig — (A) Uninfected TIVE cells were transfected with 5 nM or 10 nM concentration of siUCA1 or Scr control. At 24, 48, 72 and 96 h, the samples were subject to MTS assay and absorption was measured at 495 nm wavelength. The bar graphs show the relative absorbance ± SEM (n = 3). (B) Uninfected TIVE cells were transfected with 10 nM concentration of siUCA1 or Scr control. At 24, 48, 72 and 96 h, the samples were subject to scratch assay. Plates were imaged at 0 and 12 h and the images were processed using T-Scratch. The bar graphs show the percentage of scratch area recovered ± SEM (n = 3). For 96 h time-point, those data points where the scratch area was completely recovered were omitted. p-values: * < 0.05; ** < 0.005; and *** < 0.0005. (TIF) [file ppat.1006508.s002.tif]

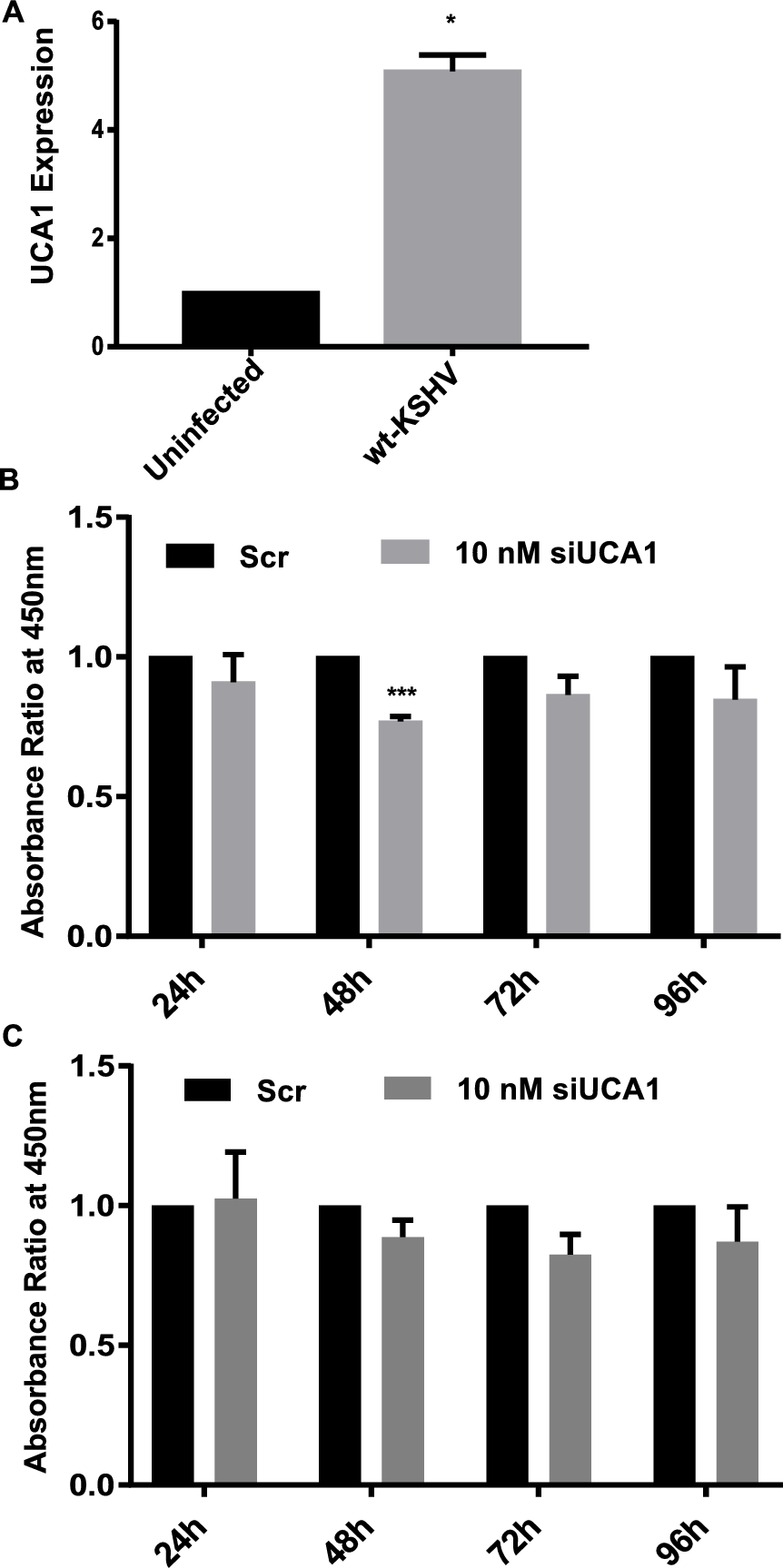

Supplement: S3 Fig — (A) UCA1 expression in uninfected and wt-KSHV-infected iSLK cells measured by qRT-PCR. The bar graphs show the mean values ± SEM after normalization to GAPDH (n = 2). (B) wt-KSHV-infected iSLK cells were transfected with 5 nM or 10 nM concentration of siUCA1 or Scr control. At 24, 48, 72 and 96 h, the samples were subject to MTS assay and absorption was measured at 495 nm wavelength. The bar graphs show the relative absorbance ± SEM (n = 3). (C) Uninfected iSLK cells were transfected with 5 nM or 10 nM concentration of siUCA1 or Scr control. At 24, 48, 72 and 96 h, the samples were subject to MTS assay and absorption was measured at 495 nm wavelength. The bar graphs show the relative absorbance ± SEM (n = 3). p-values: * < 0.05; ** < 0.005. (TIF) [file ppat.1006508.s003.tif]
