# Supplementary material for: microRNA dependent and independent deregulation of long non-coding RNAs by an oncogenic herpesvirus
Source: PLoS Pathog. 2017 Jul 17;13(7):e1006508. doi: 10.1371/journal.ppat.1006508 (PMC5531683; doi:10.1371/journal.ppat.1006508)
Supplement: S4 Table — (PDF) [file ppat.1006508.s008.pdf]

Table S4

Forward and reverse primer sequences for qPCR.		
**qPCR primers**		
**Gene**	**Direction**	**Sequence (5' to 3')**
GAPDH	Forward	CCCCTGGCCAAGGTCATCCA
	Reverse	ACAGCCTTGGCAGCGCCAGT
CD27-AS1	Forward	ACAGAATGAGTAGCAGCAGGGG
	Reverse	TGTTTCTGCCTTCCCATCCCAT
Linc00607	Forward	GACGCTGTAGGAAGAGGATTG
	Reverse	AGTAATGGTGGTGGTGGAAAC
Loc541472	Forward	TGACCTCTGTTGGGCATTTAC
	Reverse	TCGGTGAAGAATGGATGACCT
RP11-438-N16.1	Forward	TGAAGACCAGCCCAGGAATCTG
	Reverse	GGAGAAACTGAGACCGAGGAGG
MEG3	Forward	TTTTGTGCCCAAGGCTCCTGGA
	Reverse	AGGGACTCAAGGAGCCAGGTTA
ANRIL	Forward	TCTGATTCAACAGCAGAGATCAA
	Reverse	CAGCACACCTAACAGTGATGC
LSD1	Forward	CTCTTCTGGAACCTCTATAAAGC
	Reverse	CATTTCCAGATGATCCTGCAGCAA
UCA1	Forward	CTCTCCATTGGGTTCACCATTC
	Reverse	GCGGCAGGTCTTAAGAGATGAG
RNA sequences of the miRNA strand (the biotinylated strand with miRNA sequence) and the passenger strand (used to make the miRNA double stranded, but does not get incorporated into RISC). Biotin was added to the 3' end of the miRNA strand.		
**Biotinylated miRNA mimics**		
**KSHV miRNA**	**Direction**	**Sequence (5' to 3')**
miR-K12-6-5p	miRNA	CCAGCAGCACCUAAUCCAUCGUU/3Bio/
	Passenger	CGAUGGAUUAGGUGCUGCUAGAG
miR-K12-11*	miRNA	GGUCACAGCUUAAACAUUUCUAGAUU/3Bio/
	Passenger	UCUAGAAAUGUUUAAGCUGUGAUCAG
